# A Self-Powered Six-Axis Tactile Sensor by Using Triboelectric Mechanism

**DOI:** 10.3390/nano8070503

**Published:** 2018-07-06

**Authors:** Tao Chen, Qiongfeng Shi, Zhan Yang, Jinchang Liu, Huicong Liu, Lining Sun, Chengkuo Lee

**Affiliations:** 1 Jiangsu Provincial Key Laboratory of Advanced Robotics, School of Mechanical and Electric Engineering & Collaborative Innovation Center of Suzhou Nano Science and Technology, Soochow University, Suzhou 215123, China; chent@suda.edu.cn (T.C.); yangzhang@suda.edu.cn (Z.Y.); llnsun@suda.edu.cn (L.S.); 2 Department of Electrical and Computer Engineering, National University of Singapore, 4 Engineering Drive 3, Singapore 117576, Singapore; qiongfeng@u.nus.edu; 3 Center for Intelligent Sensors and MEMS, National University of Singapore, E6 #05-11F, 5 Engineering Drive 1, Singapore 117608, Singapore; 4 Hybrid-Integrated Flexible (Stretchable) Electronic Systems Program, National University of Singapore, E6 #05-4, 5 Engineering Drive 1, Singapore 117608, Singapore; 5 NUS Suzhou Research Institute (NUSRI), Suzhou Industrial Park, Suzhou 215123, China; 6 NUS Graduate School for Integrative Science and Engineering, National University of Singapore, Singapore 117456, Singapore; 7 High Technology Research and Development Center of the Ministry of Science and Technology, Beijing 100862, China; liujc@htrdc.com

**Keywords:** triboelectric nanogenerator, self-powered sensor, liquid metal, galinstan-PDMS, tactile sensor

## Abstract

Triboelectric nanogenerators (TENGs) are highly promising because they can scavenge energy from their working environment to sustainably power wearable/mobile electronics. In this work, we propose a novel and straightforward strategy for six-axis force detection and object controlling by using a self-powered sensor based on TENG. The self-powered sensor can be used in diversified tactile sensing and energy saving applications, which has been demonstrated to be able to detect normal force in the range of 0–18 N. Using the vector properties of external force, six-axis directions in three-dimensional (3D) space is detected. Additionally, it is fabricated with environmental friendly materials, i.e., galinstan and polydimethylsiloxane (PDMS), promoting its applications in more diversified situations. Because of the available and high output voltage as well as the low internal impedance, the self-powered sensor is readily compatible with commercial signal processing and management circuits. The device presented in this work shows robust structure and stable output performance, enabling itself as an ideal human machine interface in self-powered, batteryless, and electric energy saving applications.

## 1. Introduction

Due to the increasing and urgent requirement of controlling accuracy in attitude and heading fields, the design and optimization of a detection system is becoming more difficult than before, especially in multi-axis detection and application. Over the years, the main research work in these fields has been based on the mechanism of motion. However, the main problem with such a mechanism is that most devices require an external power supply. This affects the lifetime and mobility of the device. Recently, a promising self-powered technology based on triboelectric nanogenerators (TENGs) has been proposed and extensively investigated. By scavenging the mechanical energy from the surroundings, sensors based on TENGs can operate sustainably without an external power supply [1,2,3,4,5,6,7,8,9,10,11]. At present, a tactile sensor based on TENG has been widely adopted in wireless systems, robotics, biomedical fields, and portable electronics because of its high output power density [12,13,14,15,16,17,18,19,20,21]. According to the report of Cisco [22], trillions of sensors will be fabricated and distributed for various applications in the next two years. From the perspective of technology development, self-powered, intelligent, perceptive, and maintenance-free are the main directions of sensor development in the future. Therefore, a self-powered tactile sensor has attracted more and more research interest in the application of the Internet of Things (IoT).

The characteristics of TENG have received extensive research efforts across the world [23,24,25,26,27,28,29,30,31,32]. Most of the mechanical movements in daily life can generate useful energy using TENGs. In terms of tactile sensors based on TENG, a large variety of sensing models have been developed. For example, Wang’s group reports a self-powered triboelectric sensor that can accurately detect the movement of a moving object based on an array of single-electrode TENGs in two dimensions [33]. Shi et al. report a microfluidic pressure/force sensor with two sensing mechanisms for complemental detection of dynamic and static pressure [34]. Zhang’s group reports a micro-patterned polydimethylsiloxane (PDMS) tactile sensor with the feature of transparent and single-friction-surface [35]. According to TENG theory, when the tactile sensor is operating, the charges generated by triboelectrification can drive electrons to flow between different electrodes [36,37,38,39]. Four classical models are commonly used to analyze and study various situations [40]. In the single electrode mode, another electrode is normally grounded. When the active object approaches or leaves the triboelectric layer, it changes the distribution of the electric field. Then electrons flow between the electrode and ground to balance the electric field, generating electric current in the external circuit. This is the basic qualitative working mechanism of the self-powered sensors.

Currently, in the studies of tactile sensors based on TENGs, the normal force is analyzed more frequently with high sensitivity and robust performance [41,42,43]. However, the external force contains both the normal and the shear force. There are a few studies on shear force detection based on TENGs. In this work, we propose a novel strategy for six-axis force detection and object controlling using a self-powered sensor based on TENG. This sensor can be used for diversified tactile sensing and energy saving applications, with the ability to detect normal force in the range of 0–18 N. Using the vector properties of external force, six-axis directions in 3D space can be detected. The six-axis self-powered sensor exhibits robust structure and stable output performance, enabling diversified applications in wireless systems, robotics, and biomedical systems.

## 2. Materials and Methods

**Figure 1:**
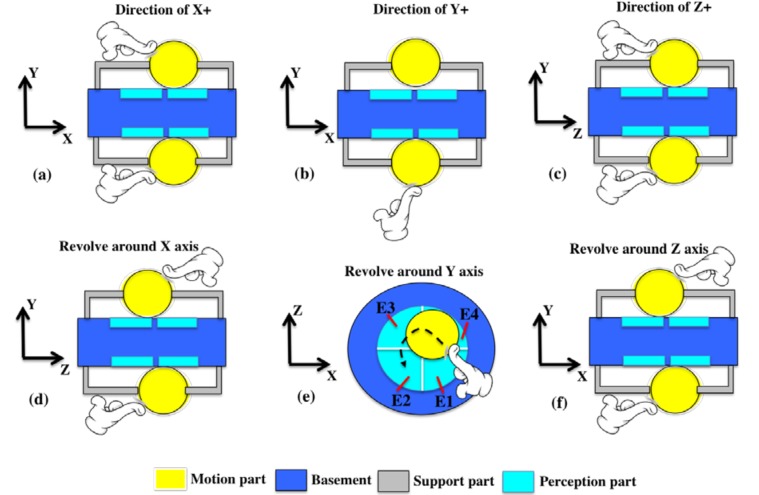
Schematic diagram of six-axis attitude detection. (**a**) In the XY plane, the fingers push both spheres to the X direction at the same time. The signals in the X direction will be detected simultaneously in the upper and lower perception part on the right side. (**b**) In the XY plane, the fingers push the sphere to the Y direction. The signals in the Y direction will be detected in the lower perception part. (**c**) In the YZ plane, the fingers push both spheres to the Z direction at the same time. The signals in the Z direction will be detected simultaneously in the upper and lower perception part on the right side. (**d**) In the YZ plane, the fingers push the spheres along the two opposite directions of the Z axis, and the signals of rotation around the X axis will be detected by the perception parts in the upper left and lower right. (**e**) In the XZ plane, the fingers push the spheres along the path shown in the figure, and the signals around the Y axis will be detected by the four perception parts in turn. (**f**) In the XY plane, the fingers push the spheres along the two opposite directions of the X axis, and the signals of rotation around the Z axis will be detected by the perception parts in the upper left and lower right.

For the six-axis attitude detection, it is necessary to detect the 3D parameters (X, Y, Z and θ_X_, θ_Y_, θ_Z_). A schematic diagram of a six-axis attitude detection is shown in Figure 1. The sphere is fixed at the middle of the device by the elastic supporting structure. Under the action of external force, the sphere is forced to contact with different perception regions (i.e., sensing electrodes), which can detect the direction of the sphere’s movement. Through combining the relative positions of the two spheres, the attitude of six degrees of freedom can be represented. The signals of these positions are detected by the perception regions, and the following six modes of motion controlling (a–f) can be realized. These six modes represent directions of the 3D movement (X, Y, Z and θ_X_, θ_Y_, θ_Z_) of an object. Therefore, this paper mainly studies how to detect these six directions of signal output.

**Figure 2:**
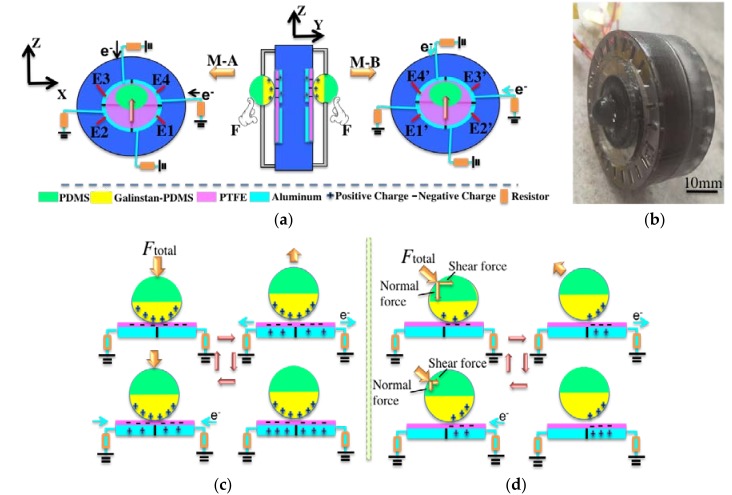
(**a**) The symmetrical structure of the self-powered sensor. (**b**) Photo of the fabricated sensor. (**c**) The working mechanism of the six-axis self-powered sensor under normal force. (**d**) The working mechanism of six-axis self-powered sensor under shear force.

The six-axis self-powered sensor is composed of two identical modules (M-A and M-B) as shown in Figure 2a. These two modules are assembled symmetrically, with a photograph depicted in Figure 2b. Based on the theory of TENG, a 3D TENG structure is fabricated with a galinstan-PDMS sphere and a PTFE layer as the triboelectric contacting surfaces. The working mechanism of each module under normal force is shown in Figure 2c. The working mechanism under shear force is shown in Figure 2d. It can be seen from the cross sectional view that each module includes the top semi-sphere, bottom semi-sphere, supporting membrane (made by PDMS) of the sphere, PTFE thin layer, Aluminum (Al) electrode layer and the housing structure. The bottom semi-sphere is fabricated with the mixture of galinstan and PDMS. The galinstan-PDMS mixture is a good conductive material which acts as a movable part and positive triboelectric material in the operation process. The Al layer is divided into four electrodes (E1–E4) to facilitate the identification and detection of the directions in the coordinates.

The two contacting surfaces (i.e., Galinstan-PDMS mixture and PTFE) are stacked face to face. After contacting with each other under external force, surface charges with opposite polarity are generated on two surfaces. When the two surfaces are separated by releasing of the external force, a small air gap is formed between them. Thus, an electric field is formed between the two electrodes. If the electrode is connected to the ground through a resistor load, the electrons will flow from the electrode to the ground. Then a reverse electric field is formed to balance the first electrostatic field. When the gap between the two friction layers is reduced under external force, the electric field formed by the triboelectric charges disappears and then electron reflux occurs. The galinstan-PDMS mixture and PTFE are chosen as the contacting layers due to their large difference of the work function. This is the basic working mechanism of the sensor under general force. For the characterization of the sensor, the normal force and shear force will be produced simultaneously when the general force acts on the top semi-sphere. The normal force presses the bottom semi-sphere to contact with PTFE, causing the output generation on the sensing electrodes. The shear force compresses and extends the PDMS supporting membrane along the shear force direction, resulting in output generation on the electrodes in that direction. When the external force is revoked, the PDMS supporting membrane pushes the whole sphere back to its original position. By analyzing the value of the output voltages on the four electrodes, the direction of the shear force can be determined. The X axis and Y axis coincides with the two borders of the E4 sector, respectively, as shown in Figure 2a.

**Figure 3:**
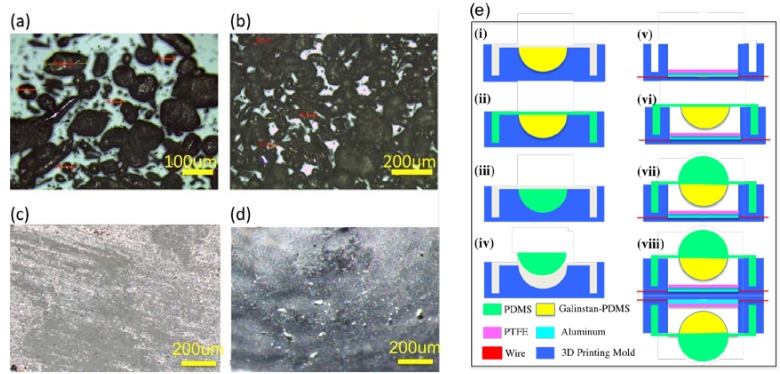
Structure of galinstan-polydimethylsiloxane (PDMS) mixture and fabrication process of the sensor. (**a**) Optical microscope image of the monolayer structure of galinstan and PDMS mixture. (**b**) Multilayer structure of the mixture. (**c**) Microscope image of the conductive layer on the semi-sphere surface under an external force. (**d**) Microscope image of the semi-sphere surface when the external force is removed. (**e**) Fabrication process of the six-axis self-powered sensor.

Prior to the fabrication of the sensor, the mixture of the galinstan and PDMS is prepared and characterized first. To investigate the surface topography of the galinstan-PDMS mixture, optical microscopy (Leica DM750, Leica Microsystems, Heerbrugg, Switzerland) is adopted. Figure 3a is the monolayer structure of the galinstan-PDMS mixture. After mixing, the galinstan particle diameter ranges from 10 to 200 μm. These particles are still in the liquid phase. Figure 3b is a multilayer structure of the mixture. The galinstan liquid droplets spread out of PDMS and form a conductive sheet when the mixture is pressed against PTFE. The conductive sheet contributes significantly to the triboelectrification as the positive friction material. In order to observe the conductive layer visually, a glass slide is used to press the semi-sphere structure made of PDMS and galinstan mixture. As shown in Figure 3c, a continuous galinstan layer on the surface can be observed. When the external force is removed, PDMS semi-sphere restores to its original shape and most of the galinstan is absorbed into the PDMS matrix, as shown in Figure 3d. Meanwhile, the excellent wetting property of galinstan on the PDMS surface and the low wettability on the PTFE surface enables galinstan to stay reliably on the PDMS surface without residue on the PTFE surface, no matter how large the pressing force is.

Based on the theoretical analysis and the mechanism analysis of multi-dimensional force detection, the design and fabrication of the self-powered sensor are then carried out. The detailed fabrication process is shown in Figure 3e. (i) Firstly, pure PDMS solution is mixed with the cross-linker. The weight ratio of them is 10:1. Then the prepared solution of PDMS and cross-linker is further mixed with galinstan. The ratio of the two materials is 1:1 by volume (6.44:1 galinstan to PDMS by weight) [44]. The bottom semi-sphere mold is made by 3D printing in advance. Then, the mixture of galinstan-PDMS is poured in the mold to fabricate the bottom semi-sphere body. (ii) Then the pure PDMS is poured in the mold to form the supporting structure. Next, degassing of the structure is carried out, followed by curing at 70 °C in an oven for 60 min. (iii) Next, pure PDMS is poured into another 3D printing mold to form the top semi-sphere. (iv) After the structure is degassed and cured, the top sphere is taken out of the mold. (v) The housing structure of the sensor is also fabricated with 3D printing. The PTFE layer and Al electrode layer are placed on the bottom of the structure. (vi) The fabricated structure with bottom semi-sphere is then demolded and assembled into housing structure. (vii) PDMS top semi-sphere is bonded on the bottom of the semi-sphere by PDMS-PDMS bonding method. (viii) Two modules are attached together back to back to form the complete sensor. The dimensions of the sensor are shown in the Table 1.

## 3. Results and Discussions

### 3.1. Detection of Normal Force 

**Figure 4:**
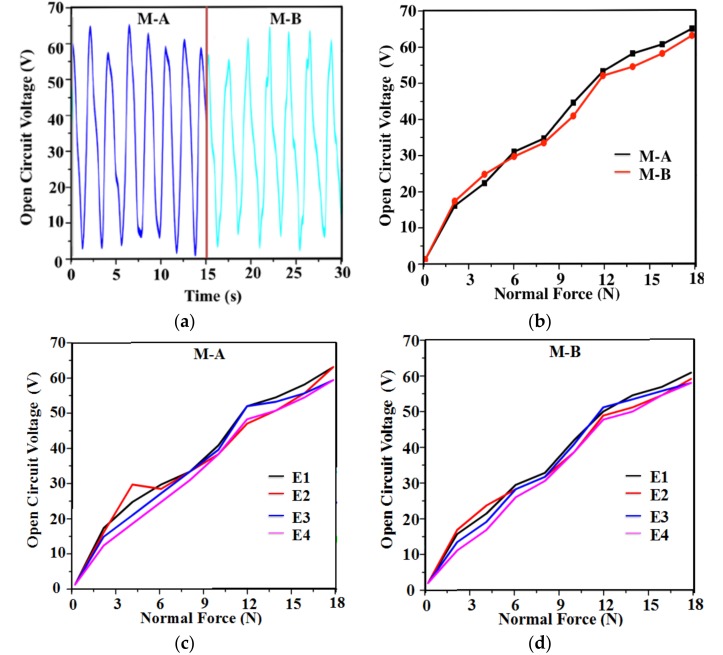

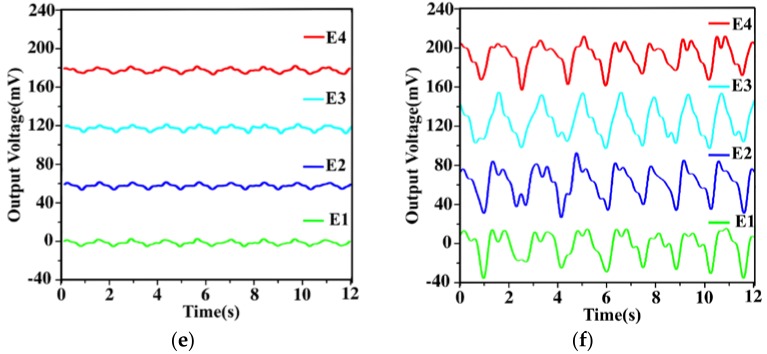
(**a**) The open circuit voltage under the force of 18 N. (**b**) The relationship of open circuit voltage with applied normal force. (**c**) The open circuit voltage on E1–E4 electrodes (M-A). (**d**) The open circuit voltage on E1–E4 electrodes (M-B). (**e**) The output voltage on the four electrodes under normal force of 1.5 N. (**f**) The output voltage on the four electrodes under the normal force of 10 N.

The shear force has a proportional relationship with the normal force, and is determined by normal force. Because the six-axis self-powered sensor composed of two identical modules, each module will be characterized for normal force sensing, with the help of a force gauge testing system. The hammer of the force gauge is set to contact with the top semi-sphere without any deformation. As the radius of the sphere is 5 mm, the displacement of the hammer movement is set to 5 mm to ensure that the PTFE film is fully charged. The number of the moving cycle is set to 50. Figure 4a shows the output performance of module A and module B, respectively.

It can be seen from Figure 4a that the maximum open circuit voltage reaches 65 V. As shown in Figure 4b, the relationship of the output voltages and the applied force is presented. In the force range from 0 N to 18 N, the outputs show good linearity and are useful in the application of self-powered sensing. The sensitivity of open circuit voltage for normal force detecting is 3.6 VN^−1^. The open circuit voltage output of E1–E4 in M-A and M-B at 0–18 N is shown in Figure 4c,d. The output voltages of E1–E4 at 1.5 N and 10 N are shown in Figure 4e,f. Compared with the single electrode device, the output from E1–E4 show similar linear characteristics and output amplitude.

### 3.2. Detection of Shear Force

To ensure the reliability of the testing results, the force is applied at the limitation of finger capability on the top semi-sphere of the sensor. Thumb and index fingers maintain the same amplitude of around 10 N and the same vertical angle α in different horizontal directions. For the sensing application, the amplitude of the output voltage of shear force is represented by

(1) $$V_{shear}=\sqrt{V_{E1}^{2}+V_{E4}^{2}}$$

**Figure 5:**
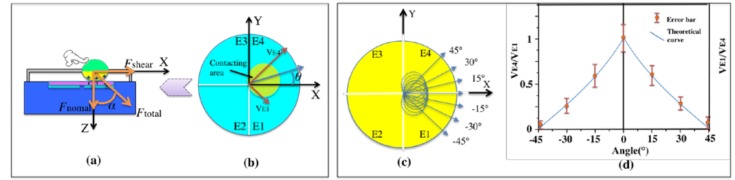
(**a**) Analysis of force component acting on the self-powered sensor. (**b**) The structure form with four electrodes and working mechanism for shear force detecting. (**c**) Repetitive motion of finger in different directions from −45° to 45°, increasing in a step of 15°. (**d**) Relationship between the measurement curve with error bars of *V_E_*_1_/*V_E_*_4_ (0–45°), *V_E_*_4_/*V_E_*_1_ (−45°−0), and the theoretical curve.

As shown in Figure 5b, the horizontal angle θ, between the x-axis positive direction and the shear force, is derived by

(2) $$\theta =45{}^{\circ}-tan^{-1}\frac{V_{E1}}{V_{E4}}$$

In order to detect the resolution of the in-plane angle θ, repeated motions of finger are applied in different directions, with the angle increasing in a step of 15°. Figure 5d shows the 7 repeated motions along the directions of −45°, −30°, −15°, 0°, 15°, 30°, and 45°, respectively. The error bars of the 7 motions have no overlapping area in the projection of ordinate. Therefore, the angle resolution of shear force detection is defined as 15°. In Figure 5b, the angle θ is defined as the acute angle between the positive x-axis direction and the shear force direction. Thus, the ratio in Equation (2) is changed to *V_E_*_4_/*V_E_*_1_ when analyzing the negative degrees (0 to −45°). Therefore, the result shown in Figure 5d is bilateral symmetry at 0°, and the vertical axis is *V_E_*_1_/*V_E_*_4_ and *V_E_*_4_/*V_E_*_1_, respectively. When the angle exceeds −45° or 45°, the calculation will be changed to the next pair of electrodes correspondingly.

**Figure 6:**
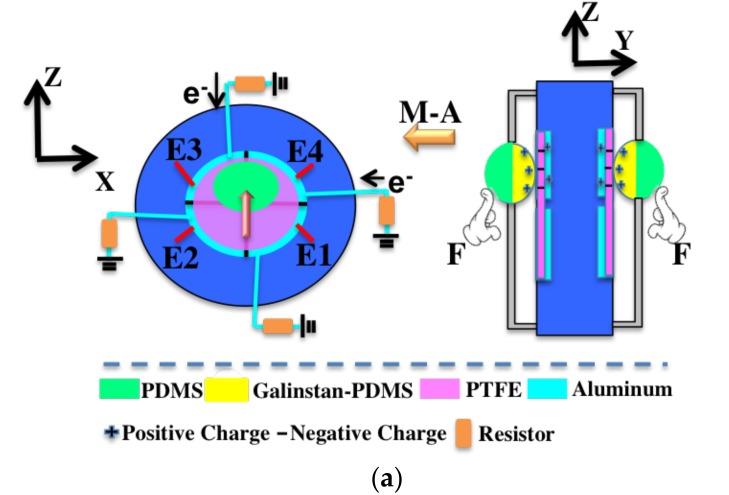

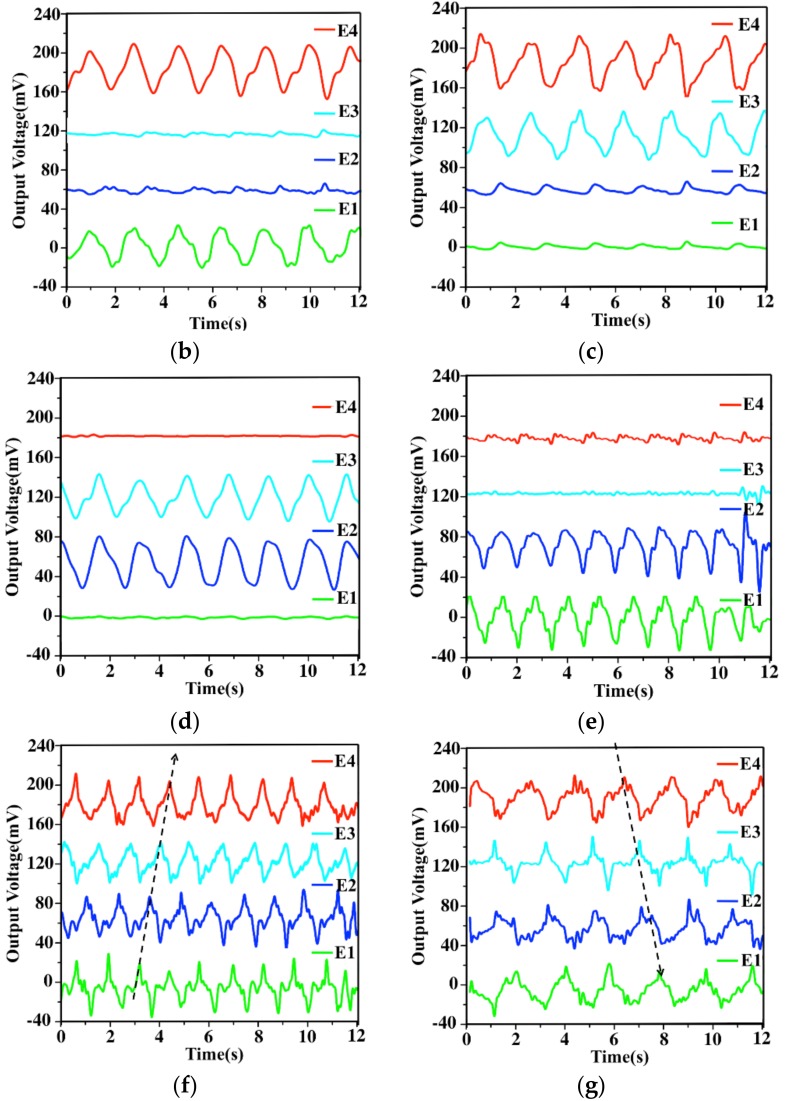
(**a**) The structure of module A (M-A) and the distribution of electrodes (E1–E4). (**b**–**e**) Output voltage waveform of M-A by repetitive motions in 0°, 90°, 180°, 270° directions. (**f**,**g**) Detection of the rotation direction around Y axis.

Similar to the detection of normal force, the characterization of shear force sensing of the sensor will also be conducted for each module. To ensure the reliability of the testing results, the force acting on the semi-sphere of the sensor is kept at the same amplitude of 10 N and the same horizontal angle. Firstly, the shear force detection along the direction of the X axis and Y axis (0°, 90°, 180°, 270°) is performed, as shown in Figure 6a–d. In order to improve the accuracy and reliability of the detection, the finger on the sphere moves repeatedly for more than 5 cycles. The average peak values of output voltages are calculated from the generated signals. It can be seen that the direction of X axis and Y axis can be accurately distinguished through the signals of the four electrodes.

According to the principle of the six-axis attitude detection, finger rotating the top semi-sphere around the center is conducted. Figure 6e,f shows the outputs of the rotation motion. In Figure 6e, there is a 0.5 s time delay from E1, E2, E3 to E4, indicating that the movement is clockwise. Figure 6f shows that the rotation action is counterclockwise. The measurement result demonstrates that the self-powered sensor can detect the rotation direction of the finger movement. The detection of rotation around the Y axis as shown in Figure 1 is realized.

By the vector decomposition of the forces acted on the spheres, the normal and shear forces on the two modules can be obtained. Through the combinational detection signals of the normal and shear forces, the 3D attitude detection can be realized with parameters (X, Y, Z and θ_X_, θ_Y_, θ_Z_) in the rectangular coordinate system.

### 3.3. Characterization of Six-Axis Attitude Detecting

In 3D space, the transformation between different coordinate systems is the transformation of the different original points and coordinate axes. The rotation of the two coordinate systems can be regarded as a coordinate system that rotates three times to another coordinate system, and the three rotation angles are Euler angles. Because the translation and scale transformation are simple, the rotation transformation in the rectangular coordinate system is considered. Therefore, the attitudes of an object in 3D space can be characterized by the six-axis parameters (X, Y, Z and θ_X_, θ_Y_, θ_Z_) in the rectangular coordinate system.

**Figure 7:**
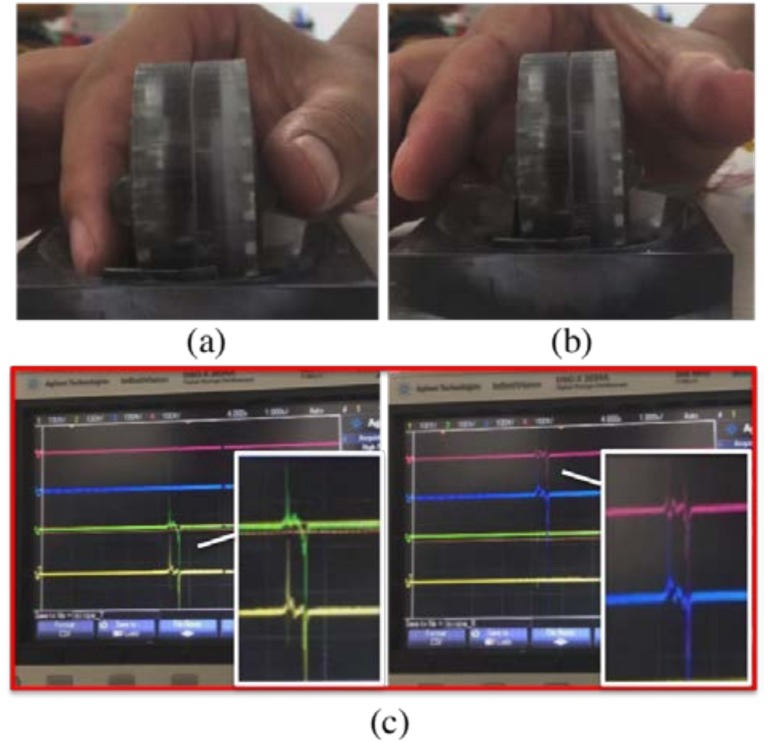
(**a**) Photograph of the thumb and index finger pressing the two spheres. (**b**) The two fingers releasing from the spheres. (**c**) The corresponding signals display on the oscilloscope.

In view of the above sections, the normal force and shear force of each module are tested respectively. In order to realize six-axis attitude detecting, a strategy combined the component vector is proposed. The testing of using the sensor to realize the attitude detection is shown in Figure 7. The thumb and index finger press the two spheres, and the signals are collected and analyzed by two oscilloscopes.

The triboelectric sensing is based on the principle of contact-separation mode. Therefore, the detection and control of the self-powered six-axis sensor mainly use the pulse period characteristic of the detected signals. In the processing and application of the detected signals, each pulse corresponds to the unit distance and unit angle on the six axes. The actual distance and angle values are set by the application program. Table 2 shows the relationship between the outputs of the 8 electrodes and six-axis attitude. In order to achieve accurate attitude operation and avoid the influence of signal deviation and noise, a threshold “1” is set for the voltage output under conventional force operation. The threshold value is slightly lower than the minimum peak value of the corresponding electrodes aimed at the specific applications.

**Figure 8:**
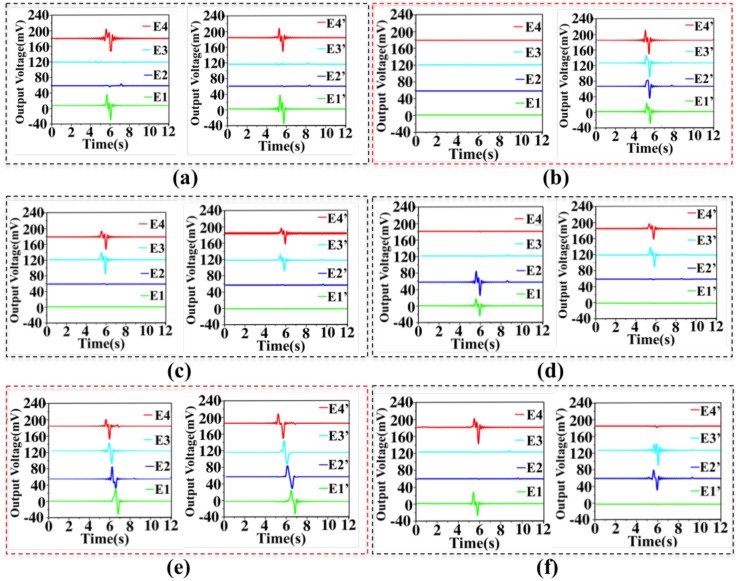
The operating mode and voltage output of the sensor. Voltage output of sensor corresponding to moving along X direction (**a**), Y direction (**b**), Z direction (**c**) and revolving around the X axis (**d**), Y axis (**e**), Z axis (**f**), respectively.

According to the attitude in 3D space, the normal forces and shear forces detected by the eight electrodes simulate the space vector (X, Y, Z and θ_X_, θ_Y_, θ_Z_). The eight signals of the electrodes are simultaneously detected and analyzed as shown in Figure 8. Every group with two pictures corresponds to one movement mode. To better distinguish four electrode outputs in the diagram, E2(E2′), E3(E3′) and E4(E4′) are set to have a positive voltage offsets of 60 mV, 120 mV and 180 mV, respectively. Figure 8a–f depicts the voltage values of the sensor for moving along X, Y, Z direction and revolving around the X, Y, Z axis, respectively. As mentioned above, the voltage greater than the threshold is set to be “1”. As shown in Figure 8a, when the sensor indicates the direction of X+, (E1, E4) and (E1′, E4′) increase to the values larger than the threshold because the contacting areas of the two spheres are all on E1, E4 and E1′, E4′. Similarly, values of (E2, E3) and (E2′, E3′) larger than the threshold indicate the direction of X−. The representation of the Z direction is the same as the X direction. The larger values of (E3, E4) and (E3′, E4′) indicates the direction of Z+ as shown in Figure 8c. The larger values of (E1, E2) and (E1′, E2′) indicates the direction of Z−. The representation of the Y direction is different from the X direction. The two symmetric modules control Y+ and Y− separately. When the four electrodes (E1, E2, E3, E4) have the same value, it indicates the direction of Y−. Meanwhile the four electrodes (E1′, E2′, E3′, E4′) have no signals. When the four electrodes (E1′, E2′, E3′, E4′) have the same value, it indicates the direction of Y+ as shown in Figure 8b. For the rotation sensing, the larger values of (E1, E2) and (E3′, E4′) indicates the counter clockwise rotation attitude θ_X_+ as shown in Figure 8d. The largest values of (E3, E4) and (E1′, E2′) indicates the clockwise rotation direction θ_X_−. The representation of the θ_Z_ direction is the same as θ_X_ direction as shown in Figure 8f. To indicate the θ_Y_, the two fingers pinch the spheres at the same time, and then spin clockwise (or counterclockwise) around the Y axis. The delay from E4, E3, E2 to E1 (E4′, E3′, E2′ to E1′) indicates a counterclockwise rotation of θ_Y_+ as shown in Figure 8e. If the delay is in the opposite direction, then it indicates a clockwise rotation of θ_Y_−.

It can be seen that the sensor can clearly identify the generated signal from each electrode. At the same time, the six-axis attitude detection can be achieved through a combination analysis of these signals. These data can be processed and programmed by a system to control the six degrees of freedom of an object in 3D space.

## 4. Conclusions

In summary, a six-axis self-powered sensor is proposed and investigated. Based on the triboelectric mechanism, it can work as a tactile sensor by using the symmetric structure. The self-powered tactile sensor can accurately detect both the normal force and shear force, which can be further used to realize the six degrees of freedom controlling. The device shows good linearity in normal force sensing, which is useful in the application of self-powered sensing. The voltage sensitivity of normal force detection is 3.6 VN^−1^. The symmetric sensor module consists of eight sensing electrodes. The characteristics of the normal force and the shear force are calibrated by voltage values of eight electrodes. The combination of the eight scalars simulates the space vector (X, Y, Z and θ_X_, θ_Y_, θ_Z_). Therefore, the sensor is able to realize 3D attitude detection and object controlling, showing great potential in diversified self-powered sensing and controlling applications.

## Figures and Tables

**Table 1:** Dimensions of the sensor.

Parameter Name	Value
Radius of sphere	5 × 10^−3^ m
Radius of electrodes	10 × 10^−3^ m
Gap between electrodes	1 × 10^−3^ m
Spacing between PTFE and sphere	2 × 10^−3^ m
Diameter of integral sensor	50 × 10^−3^ m
Thickness of integral sensor	32 × 10^−3^ m

**Table 2:**
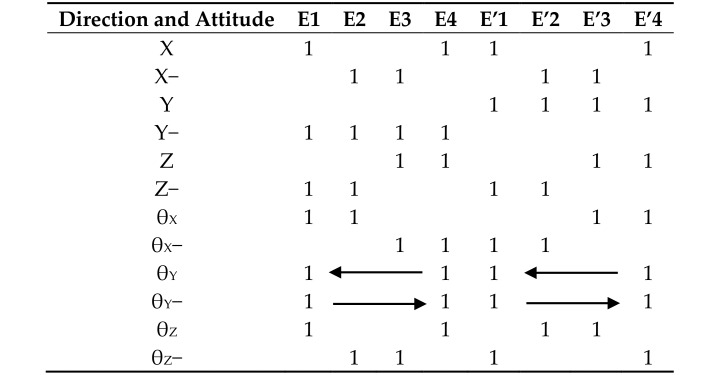
Output of the electrodes corresponding to the different operation instructions.
